# Identification of Viral Signatures Using High-Throughput Sequencing on Blood of Patients With Kawasaki Disease

**DOI:** 10.3389/fped.2019.00524

**Published:** 2019-12-19

**Authors:** Arnaud G. L'Huillier, Francisco Brito, Noemie Wagner, Samuel Cordey, Evgeny Zdobnov, Klara M. Posfay-Barbe, Laurent Kaiser

**Affiliations:** ^1^Division of Pediatric Infectious Diseases, Department of Pediatrics, University Hospitals of Geneva & Faculty of Medicine, University of Geneva, Geneva, Switzerland; ^2^Laboratory of Virology, Division of Infectious Diseases and Division of Laboratory Medicine, University Hospitals of Geneva & Faculty of Medicine, University of Geneva, Geneva, Switzerland; ^3^Swiss Institute of Bioinformatics, Geneva, Switzerland

**Keywords:** Kawasaki disease, etiology, high-throughput sequencing, viral infection, virus

## Abstract

**Aims:** Kawasaki disease is an acute pediatric vasculitis whose etiology remains unknown but epidemiology and clinical presentation suggest a viral etiology. We performed unbiased high-throughput-sequencing on blood of patients with Kawasaki Disease (KD).

**Materials and Methods:** High-throughput-sequencing was performed directly on blood of children with typical KD. Sequences were aligned against a database of clinically relevant viruses.

**Results:** Four patients were acutely infected in the blood, with respectively, poliovirus (vaccine strain), measles (vaccine strain), rhinovirus and bocavirus. Patients with poliovirus and measles had received oral polio and measles vaccines, respectively, twelve and 2 weeks prior.

**Conclusion:** Viral signatures were identified in more than half of the patients, including some corresponding to their vaccinal history. This could suggest a temporal association with KD.

## Introduction

Kawasaki disease (KD) is an acute vasculitis typically occurring in children aged 6 months to 5 years. Because of coronary aneurisms that can develop during the acute phase of the disease, KD is a leading cause of acquired pediatric heart disease (1, 2). KD diagnosis criteria are fulfilled in patients with at least 5 days of fever and ≥ 4/5 of the following criteria: bilateral conjunctivitis without exsudate, unilateral cervical lymphadenopathy, polymorphous exanthema, extremities modifications (edema, peeling, redness), and changes in the lips and/or oral cavity (erythema, strawberry tongue) (1, 3).

Despite extensive research, KD's etiology is still unknown. Epidemiological and clinical characteristics such as the high-grade fever, elevated acute-phase reactants and white blood cell count strongly suggest an infectious cause (1). Moreover, the rarity of KD after 5 years and the almost absence of recurrence suggests the development of protective immunity; similarly, the rarity of KD before 6 months suggests passive protection through maternal antibodies. The seasonal pattern, the occurrence of outbreaks, the familial clusters and the response to intravenous immunoglobulins also suggest an infectious cause (4–6). Some characteristics more specifically suggest a viral etiology, such as the self-limited characteristic of the disease, the polymorphous exanthema, the bilateral conjunctivitis without exudate, and some KD complications (arthralgia, elevated liver function tests, myocarditis, and aseptic meningitis) (1). As complications occur either during KD or immediately after, it is more likely that KD is an infectious entity rather than a parainfectious event, where complications would be expected to occur later.

However, extensive microbiological investigations, such as cultures and serologies reported inconsistent results and failed to identify a causative agent. In recent years, studies using unbiased screening by high-throughput sequencing (HTS) have led to significant advances by confirming viral infections as the cause of clinical syndromes with previously unknown etiology. Given the fact that KD is a vasculitis and its systemic presentation, we hypothesized that, if KD is associated with viruses, the causative agent should be found in blood. The aim of our study was to investigate the potential viral etiology of KD using HTS on blood specimens of children presenting with KD.

## Methods

### Clinical Specimens

Patients 6 months to 5 years old diagnosed with typical KD between January 2014 and February 2016 were prospectively enrolled. Diagnosis of KD was made by a staff pediatric emergency room physician using published KD criteria (1). Blood was collected before intravenous immunoglobulins administration. Written informed consent was obtained from parents before blood collection. The study was approved by the institutional review board. Written informed consent was obtained from parents before blood collection. HTS analysis was done in 2016.

### High-Throughput Sequencing and Sequences Analysis

For each sample, 220 ul of plasma (P1, P2, P4, and P5) or serum (P3, P6, and P7) were centrifugated at 10,000 × g for 10 min. Two-hundred ul of cell-free supernatant were treated with 40 U of Turbo DNAse (Ambion, Rotkreuz, Switzerland). Viral nucleic acids were extracted according to the RNA and DNA protocols previously published (7). The RNA and DNA libraries were prepared using the TruSeq total RNA preparation (Illumina, San Diego, US) and the Nextera XT (Illumina) protocols, respectively (7). Libraries concentrations and size distributions were evaluated with the Q-bit (Life Technologies, Carlsbad,CA, USA) and the 2200 TapeStation (Agilent, SantaClara, CA, USA), respectively. HTS (paired-end sequencing using the 100-bp protocol with indexing on a HiSeq 2500 (Illumina) raw data were analyzed using an updated version of the ezVIR pipeline as previously described for the identification of mammalian viruses (7). Briefly, it filters out low quality reads, host genome, and low complexity reads before aligning the data against a database of clinically relevant viruses. Cross-talk between libraries in the same lane was checked by applying a ratio of 0.4% (8). Of note, DNA library sizes varied between 25.088.396 and 51.935.451 reads (average: 34.178.949, median: 30.492.745), and RNA library sizes varied between 28.801.729 and 63.577.993 reads (average: 43.225.670, median: 36,927,226) (Supplementary Table 1).

### Data Availability

The raw sequences data were deposited in the National Center for Biotechnology Information (NCBI) Sequence Read Archive (SRA) under BioProject accession number PRJNA589017 (https://www.ncbi.nlm.nih.gov/bioproject/589017).

## Results

### Patients Characteristics

Seven patients with typical KD were enrolled. All patients were previously healthy with no relevant medical history. Median age was 17.1 months (interquartile range: 21.0) and 57.1% (4/7) were female. Four (57.1%) patients had four KD criteria, with patient (P)3 having coronary dilatation on echocardiography. The remaining three patients had 5 KD criteria (Table 1). Conjunctivitis and cheilitis were present in all patients, exanthema in 6 (85.7%), hand/foot edema/peeling in 6 (85.7%), and unilateral cervical adenopathy in 5 (71.4%) (Table 1).

**Table 1:** Distribution of Kawasaki disease criteria and days of fever among study patients.

	**P1**	**P2**	P3^*^	**P4**	**P5**	**P6**	**P7**	
**DEMOGRAPHICS**								
Age, months	13.3	35.2	10.5	9.9	17.1	31.0	31.4	
Gender	F	F	M	M	M	F	F	
Days of fever until Kawasaki diagnosis	5	5	10	5	8	5	5	
								**Total number of KD criteria**
**KAWASAKI DISEASE CRITERIA**								
Cheilitis	✓	✓	✓	✓	✓	✓	✓	7
Conjunctivitis	✓	✓	✓	✓	✓	✓	✓	7
Exanthema	✓	✓		✓	✓	✓	✓	6
Hand/Foot edema/peeling	✓	✓	✓	✓		✓	✓	6
Unilateral cervical adenopathy	✓	✓	✓	✓	✓			5

*P, Patient; F, Female; M, Male; KD, Kawasaki disease*.

* *P3 had evidence of coronary dilatation*.

### HTS Results

We recovered an average of 19,557,665 non-human read-pairs for DNA libraries (60.15%) and 3,216,215 read-pairs for RNA libraries (6.8%) (Supplementary Figure 1, Supplementary Table 1). From these, an average of 65'678 (0.29%) were identified as viruses.

**Figure 1:**
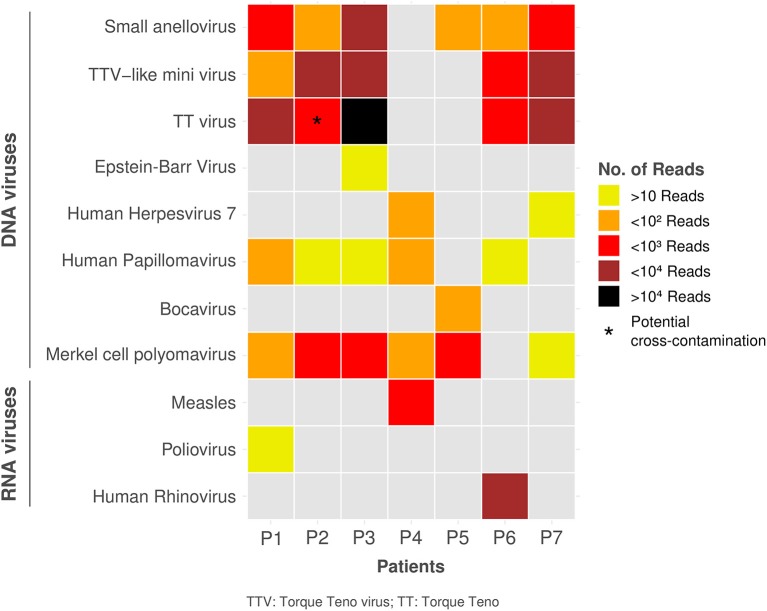
Distribution of identified viruses with number of reads among study patients.

The most frequently found viral sequences were small anelloviruses (*n* = 6, 86%, average coverage: 8.6%) and merkel cell polyomavirus (MCPyV) (*n* = 6, 86%, average coverage: 43.3%), followed by human papillomavirus (HPV) (*n* = 5, 71%, average coverage: 5,3%), Torque Teno Virus (TTV)-like mini viruses (*n* = 5, 71%, average coverage: 8.6%) and TTV (*n* = 4, 57%, average coverage: 22.8%) (Figure 1). Measles Schwartz-FF8 vaccinal strain was detected in one patient (23.8% coverage) who received an MMR vaccine 13 days prior to admission. Poliovirus Sabin-3 vaccinal strain was also detected in one patient (19.7% coverage) who received an oral polio vaccine (OPV) in Brazil 3 months prior to admission.

P6 was positive for Human Rhinovirus (HRV). The ezVIR phase 2 (strain-typing phase) on HRV reads revealed the presence of an HRV-C species (96.2% genome coverage).

### Polymerase Chain Reaction and Serology

Positive HTS samples with a sufficient leftover volume had specific real-time (RT)-PCR performed for EV (P1), measles (P4), bocavirus (P5), MCPyV (P1, P2, P3, P4, P5, and P7), TTV (P1 and P3), EBV (P3), and HHV-7 (P4 and P7) viruses: only bocavirus in P5, TTV in P1 and P3, and MCPyV in P1 and P5 could be detected. For P6, the ezVIR phase 2 (strain-typing phase) on Human Rhinovirus (HRV) reads revealed the presence of an HRV-C species (96.2% genome coverage), but there was no leftover specimen to perform reverse-transcription RT-PCR.

Serology for measles was performed in P4 and was negative for IgM and IgG. There was no leftover serum to perform EBV serology in P3.

## Discussion

This pilot study investigates KD etiology using HTS on blood specimens of patients with typical KD. Among the seven patients tested, at least four were infected during their episode of KD, with poliovirus, measles, bocavirus and HRV-C, respectively. The patient with the positive poliovirus result received an OPV in Brazil 3 months prior to admission. All reads mapped to poliovirus Sabin-3, confirming that the sequences found in the blood were vaccinal. This is particularly interesting knowing that viremia following OPV is usually of short duration (9, 10). Similarly, the patient with measles in the blood had an MMR vaccine 13 days prior to admission and all reads mapped to the measles Schwartz-FF8 vaccine strain. Measles viremia 2 weeks following MMR vaccine is not unexpected (11). The absence of IgM and IgG against measles <2 weeks post-vaccine is in agreement with previous data (12). Bocavirus viremia is usually not detected in asymptomatic children and therefore unlikely to represent asymptomatic carriage (13). Interestingly, bocavirus DNA has previously been detected in the blood of 9% of patients with KD (14). The HRV isolated in the blood was HRV-C, confirming that HRV-C is the only HRV specie where disseminated infections are documented (15, 16). Moreover, the coverage above 96% contributes to confirm that the patient was truly viremic. Although these results do not allow to establish causality, it suggests that these wild viruses and circulating live attenuated vaccinal viruses, or the immune reaction following infections, could contribute to their KD.

Anelloviruses, which include TTV, TTV-like mini viruses and small anelloviruses, were the most frequently identified viruses in our study. The identified viruses were highly divergent between patients, which makes a contamination very unlikely. Anelloviruses have previously been identified in many anatomical compartments of KD patients, such as serum, pharynx, and lymph nodes (17–19). Moreover, anelloviruses has been detected in the coronary arteries of 1/8 deceased KD patients, although the very closely related Circovirus-like genome was identified in 4 other KD patients (20). As anelloviruses are ubiquitous and frequently identified in the blood of healthy patients (21), their relevance in KD remains to be clarified. However, although considered as commensals, a symptomatic anellovirus primo-infection could possibly be related to KD, even though there is some data suggesting very early or *in utero* infection (21).

MCPyV and HPV were found in 6/7 and 5/7 patients, respectively. This is higher than previously published data reporting DNAemia in 22% (22) and 8–15% (23, 24). However, the detection of MCPyV and HPV by HTS should be interpreted with caution as both can be found in HTS reagents or laboratory supplies (contamination) but also possibly on the skin (data not shown). This is supported by the fact that MCPyV was confirmed by real-time PCR only for 2/5 patients. Among RNA viruses, XMuLV-related virus was found in three patients; as XMuLV is a contaminant (25), we did not consider this result as significant. The fact that some viruses such as EBV and HHV-7 were identified by HTS but not by conventional PCR highlights the potential superior sensitivity of HTS but could also be related to divergent sequences, low viral load and possibly DNA or RNA degradation related to freeze-thaw cycle.

There are limitations to this study. First, a control group could have helped to better understand the role of identified organisms in KD pathogenesis. In our opinion, including control patients with other rash/fever illness or afebrile control patients would be of limited benefit given the interpatient variability in the virome. Therefore, the only control group that would have helped interpreting our results would have been baseline specimens in our study patients, which was not feasible. Similarly, follow-up specimens might have been helpful but considering the number of patients in this pilot study and the likelihood for parents to accept invasive bloodwork without clinical indication (in a population where median age was 17 months), this was not included in the study protocol. Another limitation is the lack of negative and positive whole HTS process controls [e.g., spiked samples and non-template control (NTC)] to assess environmental and/or NGS reagents potential contaminants and HTS process efficiency, respectively. Concerning the HTS specificity and the potential report of false positive hits, Asplund and colleagues recently published an important study that provides an extensive list of known or novel contaminating viral sequences that could be expected in HTS-based virome investigations (26). Thus, such laboratory-component-associated viral sequences may explain the detection of MCPyV in P2, P3, P4, and P7 samples. One important finding was that some contaminating viral sequences were not observed in NTC due to their stochastic appearance, leading to the authors' recommendation to use a large number of NTC despite cost of sequencing. HTS overall performance (e.g., sensitivity and specificity) depends on several parameters such as the sample preparation, the algorithms and the choice of the database used to analyse HTS raw data (27). Therefore, in order to obtain an optimal HTS performance, we used a robust sample preparation protocol and a bioinformatics pipeline both previously published (7, 28, 29) and benchmarked (30), with an overall sensibility very close to that of real-time (RT-)PCR assays for most of viruses recognized of clinical significance.

In conclusion, this study was able to identify viral signatures in more than half of the patients, some corresponding to their vaccinal history, which could suggest a temporal association with the disease. Larger studies are needed to confirm those findings and establish either direct causality, or indirect parainfectious causality.

## Data Availability Statement

The datasets generated for this study can be found in the https://www.ncbi.nlm.nih.gov/bioproject/589017.

## Ethics Statement

The studies involving human participants were reviewed and approved by Regional Research Ethics Committee (CCER) of Geneva University Hospital. Written informed consent to participate in this study was provided by the participants' legal guardian/next of kin.

## Author Contributions

AL'H, KP-B, and LK designed the study. AL'H, NW, and KP-B enrolled the patients. FB, SC, and EZ performed the viral and bioinformatic analysis. AL'H, SC, and FB analyzed the data and drafted the manuscript. All authors contributed to manuscript revision, read, and approved the submitted version.

### Conflict of Interest

The authors declare that the research was conducted in the absence of any commercial or financial relationships that could be construed as a potential conflict of interest.
