# Supplementary figures and images for: Identification of Viral Signatures Using High-Throughput Sequencing on Blood of Patients With Kawasaki Disease
Source: Front Pediatr. 2019 Dec 19;7:524. doi: 10.3389/fped.2019.00524 (PMC6930886; doi:10.3389/fped.2019.00524)

Supplementary Figure 1. Description of DNA and RNA reads for each specimen tested

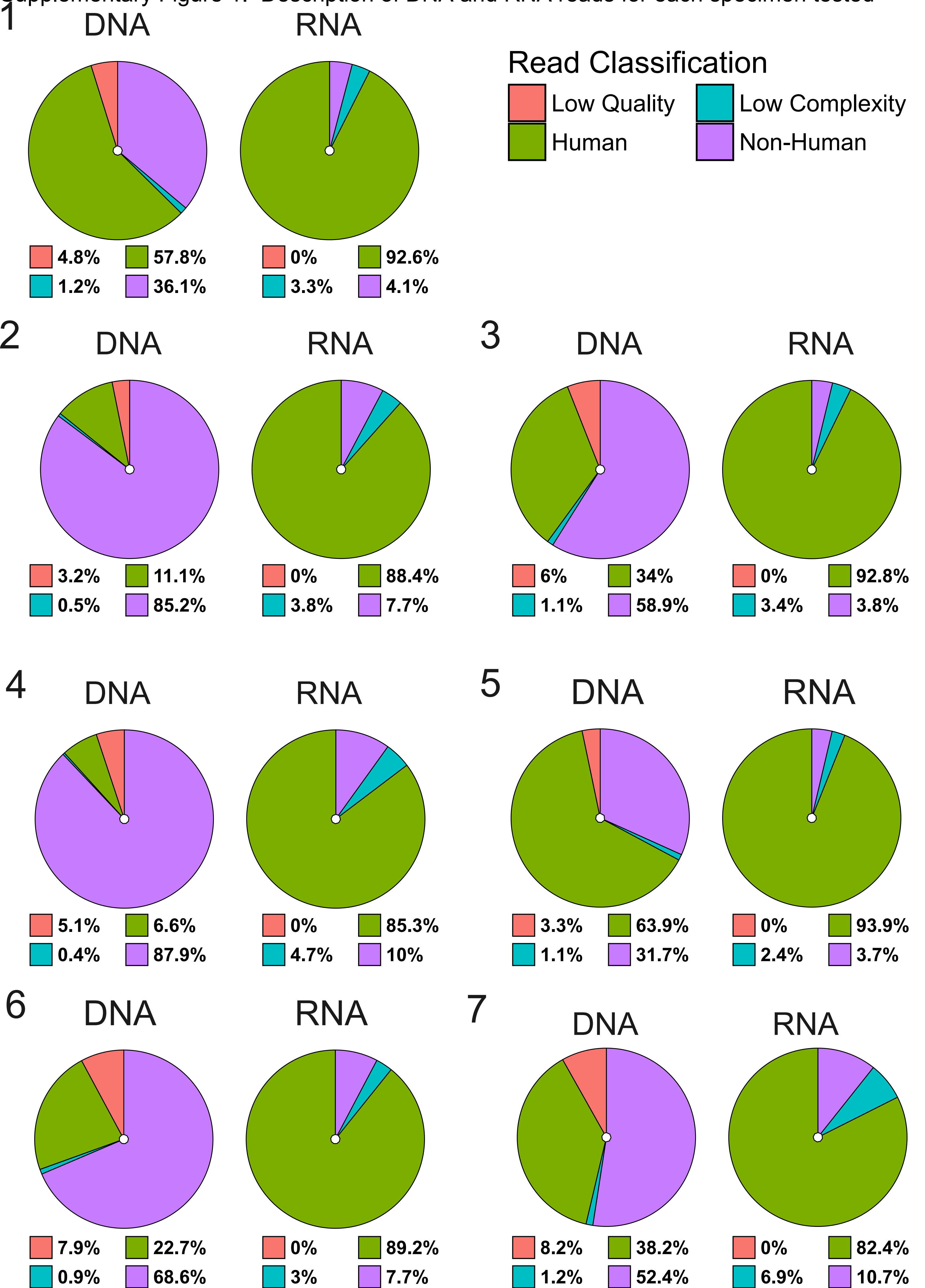

Supplement: Supplementary file 2 [file Data_Sheet_1.PDF]
